# Effective treatment of electrical storm by a wearable cardioverter defibrillator in a patient with severely impaired left ventricular function after myocardial infarction: a case report

**DOI:** 10.1186/s13256-021-02833-2

**Published:** 2021-05-17

**Authors:** Henrike Andresen, B. Sasko, D. Patschan, N. Pagonas, O. Ritter

**Affiliations:** Department of Cardiology, Brandenburg Medical School Theodor Fontane, Brandenburg an der Havel, Germany

**Keywords:** Wearable cardioverter defibrillator, Ventricular tachycardia, Post-myocardial infarction, Electrical storm

## Abstract

**Background:**

The implantation of cardioverter defibrillators (ICDs) is an established therapy in the prevention of sudden cardiac death in patients with systolic dysfunction after myocardial infarction. To avoid immediate implantation of an ICD, wearable cardioverter defibrillator vests (WCD) can be used to protect patients against malignant rhythm disorders, while at the same time drug-based heart failure therapy has to be initiated. This drug therapy can improve left ventricular ejection fraction and primary prophylactic cardioverter defibrillator implantation may not be necessary. However, the recent Vest Prevention of Early Sudden Death Trial (VEST) questioned the regular use of the WCD in this setting.

**Case presentation:**

A 47-year-old Caucasian man with severely impaired left ventricular function early after myocardial infarction was prescribed a WCD as primary prophylaxis to prevent sudden cardiac death. Seven days after the patient was supplied with a WCD, the patient suffered from an electrical storm with recurrent ventricular tachycardia (VT), which was successfully terminated 17 times by the WCD. On coronary angiography, the formerly infarct-related right coronary artery had TIMI (Thrombolysis in Myocardial Ischemia Trial) III flow, and a remaining stenosis in the left anterior descending artery (LAD) was stented, which did not stop recurrent VT. In the electrophysiology (EP) study, a focus was mapped in the left inferior ventricle, which was ablated. This stopped the VT. A second radio-frequency (RF) ablation in the same area was necessary after 14 days. Finally, a permanent cardioverter defibrillator was implanted.

**Conclusion:**

We report the case of a patient who survived recurrent episodes of VT early after myocardial infarction by effective defibrillation with a WCD. The WCD is a useful device to bridge time until a final decision for implantation of a defibrillator.

## Background

With severely impaired left ventricular function of less than 35%, the risk of life-threatening cardiac arrhythmia—ventricular tachycardia (VT) and ventricular fibrillation—increases, and thus the risk of sudden cardiac death (SCD) [1–3]. In some patients VT episodes occur as “electrical storm", defined as the recurrence of hemodynamically unstable VT over three or more episodes within 24 hours or incessant ventricular arrhythmia for more than 12 hours. The implantation of defibrillators (ICDs) is an established therapy in the prevention of SCD in patients with systolic dysfunction after myocardial infarction (MI) [1, 4]. However, the Defibrillator in Acute Myocardial Infarction Trial (DINAMIT) and the Immediate Risk Stratification Improves Survival (IRIS) trial showed that early after MI there is no survival benefit from ICD implantation [5, 6]. In the long run, by adherence to heart failure medication, an improvement in left ventricular ejection fraction can be achieved, so that primary prophylactic cardioverter defibrillator implantation is not necessarily required [7, 8]. According to current guidelines, the temporary use of a wearable cardioverter defibrillator (WCD) may be considered < 40 days after MI in selected patients (class IIb), for example patients with incomplete revascularization [9]. In line with this, a variety of registries show the efficiency in terminating VT by a wearable cardioverter defibrillator [10–12], whereas the recent Vest Prevention of Early Sudden Death Trial (VEST) did not find a significant survival benefit in patients early after MI protected by a WCD [13].

This particular case demonstrates the efficacy of the life vest concept. Additionally, we give insight into the management of electrical storm in a patient with a WCD and discuss the current recommendations on the life vest. This will also raise awareness regarding the potentially life-saving option of a WCD in selected patients.

## Case presentation

**Fig. 1:**
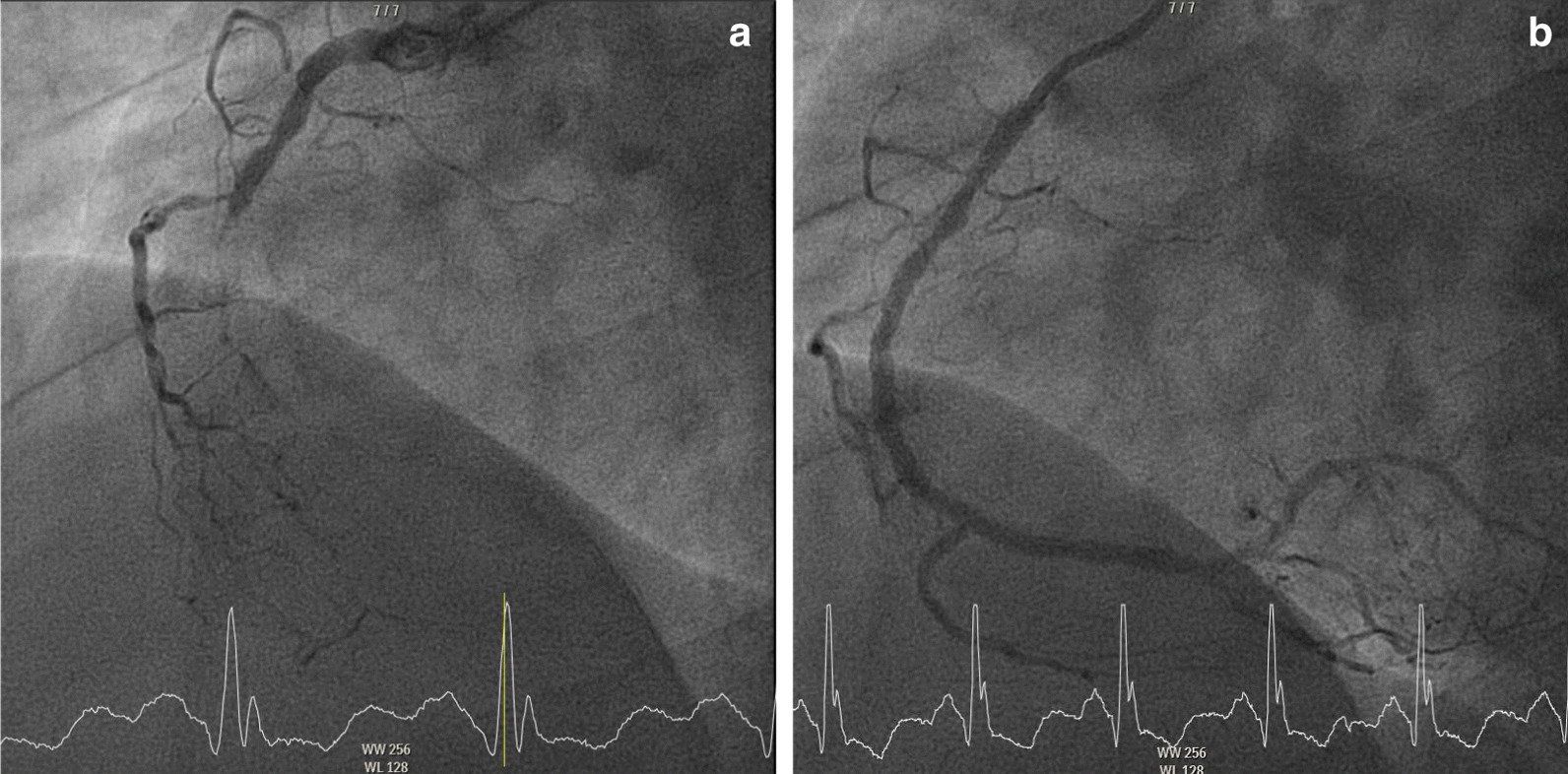
Coronary angiography. **a** Total occlusion of the right coronary artery (RCA) (left anterior oblique [LAO] view). Coronary angiography after stent implantation: **b** angiography of the RCA (LAO view)

**Fig. 2:**
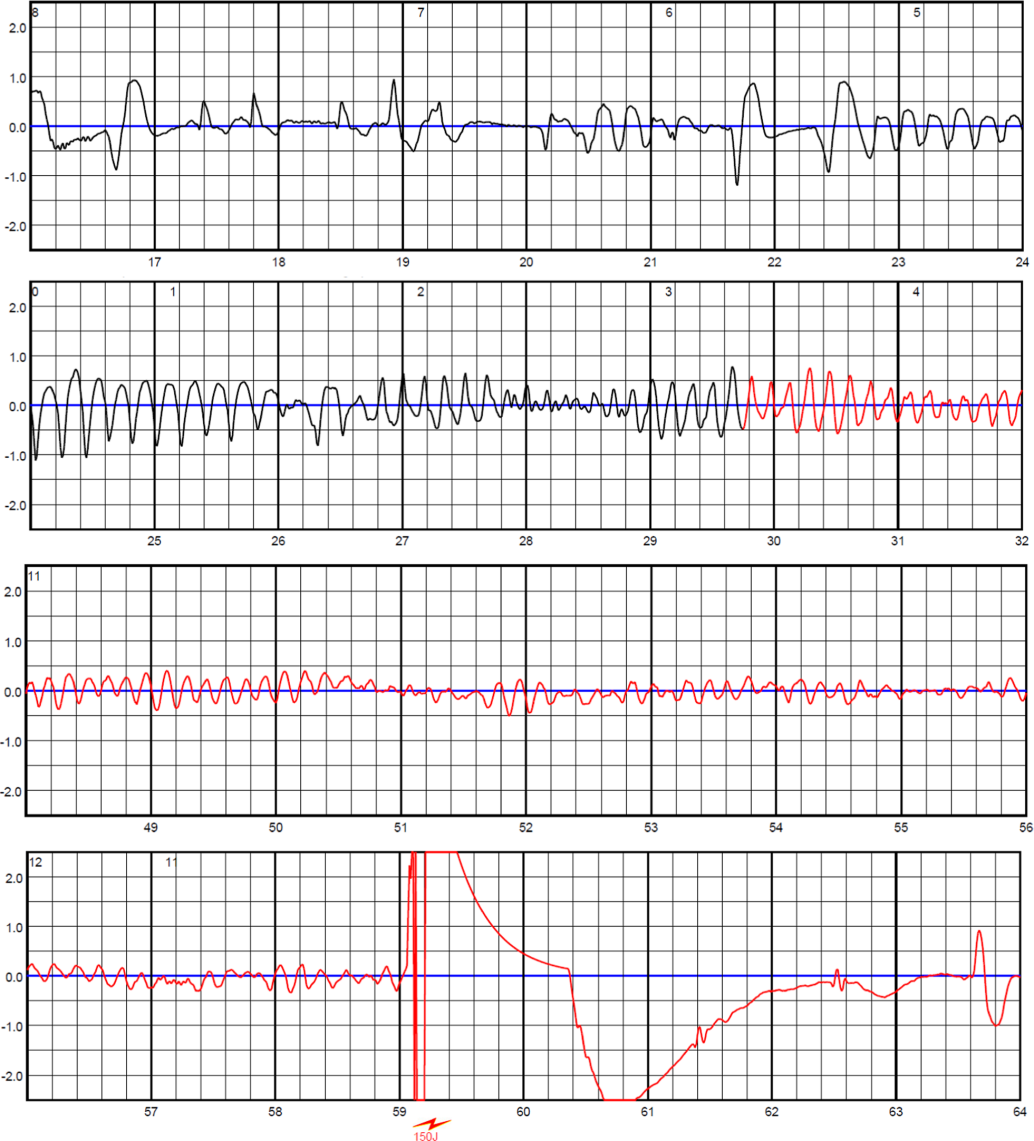
Electrocardiogram recorded ventricular tachyarrhythmia. Ventricular tachyarrhythmia is terminated by a shock 36 seconds later and subsequent sinus rhythm

A 47-year-old Caucasian man with non-ST-elevation MI was admitted by the emergency doctor to our emergency department. Apart from nicotine abuse (35 pack years), the patient had no medical history (including obsessive consumption of alcohol) or prior medication. Family, social and environmental history was unremarkable. In the emergency department, physical and neurological examination showed the following vital signs: heart rate 112 beats per minute, blood pressure 152/103 mmHg, oxygen saturation of 97% while breathing room air, respiration 15 breaths per minute, auricular temperature 37.8 °C. The patient was awake (Glasgow Coma Scale score of 15) and oriented in all respects without any neurological deficits. Electrocardiography (ECG) showed sinus tachycardia, ST-segment depressions in leads V4 and V5. He was transferred directly to the catheterization laboratory. Three-vessel disease with total occlusion of the right coronary artery (RCA), 80% stenosis of the left anterior descending artery (LAD) and 75% stenosis of the circumflex artery (Cx) was diagnosed. The RCA was stented with subsequent TIMI (Thrombolysis in Myocardial Ischemia Trial) III flow (Fig. 1). Following a “culprit lesion only” strategy, the LAD and Cx were not stented in the acute setting. Post-interventional echocardiography documented a severely reduced systolic left ventricular ejection fraction (15–20%). Besides medical therapy, which included dual antiplatelet and heart failure therapy (Table 1), the patient was provided with a WCD (LifeVest^®^, ZOLL Medical Corporation, Pittsburgh, PA, USA). He was scheduled for an early follow-up after 4 weeks. Seven days after discharge, the patient developed an electrical storm. At home, the WCD shocked twice in the evening (8:39 p.m. and 8:41 p.m.) (Fig. 2) without any prior symptoms of angina or syncope. The patient was transferred to the emergency room by ambulance. On admission, he showed normal vital signs (heart rate 88 beats per minute, blood pressure 117/81 mmHg, oxygen saturation of 99% while breathing room air, respiration 18 breaths per minute, auricular temperature 36.9 °C). He was awake (Glasgow Coma Scale score of 15) and oriented in all respects, and no neurological deficits were detected. ECG showed normal sinus rhythm, biphasic T waves in III, negative T wave in leads V5 and V6, and three ventricular extrasystoles (VES).

**Table 1:** Antiplatelet therapy and heart failure therapy on discharge

Medication on discharge after first admission			Medication on discharge after second admission and ongoing		
Medication	Dose, mg		Medication	Dose, mg	
ASS	100	One a day orally	ASS	100	Once a day orally
Ticagrelor	90	Twice a day orally	Ticagrelor	90	Twice a day orally
Ramipril	1.25	Once a day orally	Ramipril	1.25	Once a day orally
Bisoprolol	2.5	Once a day orally	Metoprolol	95	Twice a day orally
Spironolactone	25	Once a day orally	Spironolactone	25	Once a day orally

*mg* Milligram

**Fig. 3:**
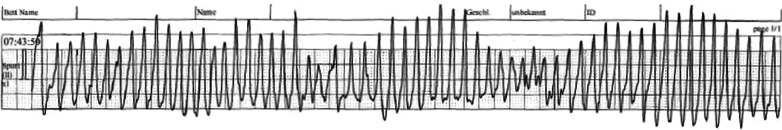
Torsade de pointes

During monitoring in the emergency room, recurrent non-sustained VT were documented and the patient received intravenous administration of 5 mg metoprolol. Initial laboratory testing revealed normal values except for increased troponin (91.1 pg/mL), without positive dynamics in a second testing (Table 2). Creatinine kinase was in the normal range as well. The patient was transferred to our chest pain unit. At night the WCD shocked again due to VT (2:56 a.m.). At no time the patient complained of angina, and there was no new evidence for recurrent myocardial ischemia in the ECG. The patient received intravenous administration of 10 mg morphine for sedation, 150 mg amiodarone and 5 mg metoprolol to suppress VT. After 4 a.m., several shocks occurred within a very short time (4:19 a.m., 4:37 a.m., 4:49 a.m., 4:51 a.m., 4:53 a.m., 4:56 a.m.), again due to monomorphic VT. The patient was sedated using intravenous 0.5 mg fentanyl, 60 mg propofol, 100 mg rocuronium, and then intubated, mechanically ventilated and transferred to the intensive care unit (ICU), where continuous amiodarone infusion (1050 mg/50 mL at 5 mL/hour) was initiated. Corrected QT (QTc) intervals were in the normal range. Within a short period of time, a second electrical storm occurred (shocks at 5:09 a.m., 5:12 a.m., 5:18 a.m., 5:20 a.m., 5:24 a.m., 5:26 a.m., 5:27 a.m., 5:30 a.m.). After additional intravenous administration of 5 mg metoprolol, the VT stopped. In the meantime, due to low battery capacity, the life vest was replaced by external defibrillator patches, and the patient was subsequently shocked another seven times. Coronary angiography was performed and two drug-eluting stents were implanted in the known 80% LAD stenosis. Despite continuous intravenous metoprolol administration (2 mg/hour) and single magnesium infusion (2 g/2 minutes), Torsade de pointes tachycardia persisted and 11 additional external defibrillations were necessary (Fig. 3).

**Table 2:** Summary of laboratory test results

Laboratory test	Result
Hemoglobin, g/L	141
White blood cells, × 10^9^/L	12.6
Platelets, × 10^9^/L	405
Troponin, pg/mL	91.1
Creatinine kinase, U/L	70
CRP, mg/dL	1.32
Serum sodium, mmol/L	141
Serum magnesium mmol/L	1,01
Serum potassium, mmol/L	4.71
Serum glucose, mmol/L	6.91
NT-proBNP, pg/mL	3026
INR	1.06
PTT, seconds	27.9
Creatinine, µmol/L	79

*NT-proBNP* N-terminal prohormone of brain natriuretic peptide, *CRP* C-reactive protein, *INR* international normalized ratio, *PTT* partial thromboplastin time

**Fig. 4:**
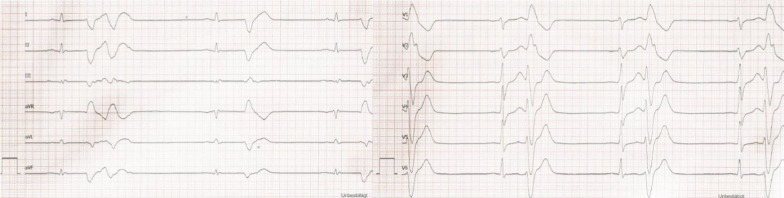
Twelve lead electrocardiogram demonstrating ventricular bigeminy

Following the coronary angiography, an EP study was performed, as the 12-lead ECGs suggested that VT were triggered by ventricular premature beats early in the repolarization phase. The premature beats appeared partly as ventricular bigeminy (Fig. 4), which induced VT with polymorphic and partially Torsade des points morphology, in some cases resulting in rapid transition into ventricular fibrillation. The focus of the VT origin/premature beats was localized in the posterior wall of the left ventricle and ablated by radio-frequency (RF) energy.

During the course of the following day, three more VT with similar morphology had to be terminated by external defibrillator shocks. Therefore, the EP procedure was repeated and the same focus was targeted by RF energy ablation. Finally, no additional VT occurred. Two days later, the patient received a dual-chamber ICD implantation for secondary prevention of sudden death. After a VT-free interval of 3 days with continuous metoprolol infusion and saturated amiodarone levels, the patient was extubated and transferred to the chest pain unit. At the end of the hospital stay, an echocardiographic follow-up of the left ventricular ejection fraction showed an improved ejection fraction of 30–35%.

During intensive care treatment, the patient suffered from ventilator-associated pneumonia which was treated with piperacillin and tazobactam intravenously for 8 days.

In a 3-month ICD follow-up by remote monitoring, there were no more shocks, but three non-sustained VT were documented.

## Discussion

We report a case of a patient with electrical storm shortly after MI. The patient was protected by a WCD, which terminated 17 episodes of repeated VT. The patient needed 18 further shocks by an external ICD until RF ablation and medical therapy established stable sinus rhythm.

In general, immediate external termination of VT is crucial for survival in the case of hemodynamic instability. The detection and treatment of ventricular tachyarrhythmia by WCDs is as effective as immediate external defibrillation [10]. WCDs can be considered for patients at risk of SCD and potential but not immediate ICD indication. Furthermore, WCDs offer patients with ICD and indication for system extraction, for example due to an active infection, an effective means of protection until a permanent ICD can be re-implanted. In this particular patient, the decision for a WCD was made because of the large infarcted area, the severely impaired left ventricular function and incomplete revascularization, since recurrent myocardial ischemia may favor ventricular arrhythmias.

Regardless of external or internal defibrillation, the relief of distress and pain in conscious patients is very important during an electrical storm. Recurrent shocks may increase sympathetic tone and therefore trigger further arrhythmias by increased levels of catecholamines. Sedation not only reduces the sympathetic activation, but also eliminates discomfort associated with invasive bedside procedures and patient management in an Intensive Care Unit (ICU) setting. Therefore, analgosedation is recommended for all patients in these situations, reducing catecholamine levels and preventing pain and further emotional distress [14]. In our case, sedation did not have an effect on the occurrence or the frequency of the VT.

As the WCD effectively sensed and terminated the recurrent VT, the vest was not taken off during the initial management of the electrical storm. Due to the automatic detection and termination of the VT, the work load for the ICU team was significantly reduced, as no team member had to be involved in terminating the rhythm disorders by shocks. However, the voice messages before each shock of the WCD were increasingly considered disturbing by the ICU team. Therefore, and due to possible low battery voltage, arrhythmia treatment was changed to external defibrillation. Of note, no burn injuries were detected on the skin.

As a routine, two batteries are given to the patient by the manufacturer. One battery is recommended to be used with the vest, while the second battery stays in the charging station. The total number of effective shocks is dependent on several factors, for example the amount of time that the battery was already in operation or the number of alarm signals. According to the manufacturer, the residual battery capacity after 24 hours is sufficient to deliver five shocks with 150 joules [15]. Although each fully charged battery provides power for more than 24 hours, our patient followed this advice and changed the battery in the morning. Therefore, our case demonstrates the capability for 17 effective shocks despite 12 hours of operating time. According to the manufacturer, there is one unpublished case of a patient with 30 effective shocks without the battery being discharged. Furthermore, due to the optimal shock vector (anteroposterior position of the patches), the shock energy may be lower in comparison to automated external defibrillators (AED with 150–360 joules) with patches in the anterolateral position [15].

Guidelines for primary prevention of sudden death recommend a waiting time of 40 days after acute MI and 90 days after coronary revascularization before permanent ICD implantation. Randomized trials have shown no benefit for ICD implantation early after acute MI [5, 6]. Nevertheless, the risk for SCD remains increased during this waiting period, despite guideline-directed medical therapy and revascularization. Therefore, an intermittent protective device might be a useful option to reduce the SCD risk during this period. However, in an intention-to-treat analysis in the recent VEST trial, the WCD therapy showed no statistically significant reduction of SCD, but indicated a trend towards a lower risk of SCD in the WCD group. Furthermore, WCD treatment was associated with lower total mortality in the first days after MI. Fourteen of 20 patients who received an appropriate shock survived longer than 90 days. In contrast, 14 diseased patients did not wear the WCD despite assignment to the treatment group. The nonadherence to wearing the device may have biased the results [13, 16]. Therefore, in an “as treated” analysis (…“if WCD would have been actually worn”…), arrhythmic death and mortality could be significantly reduced [17]. Focusing on patient comfort, one trial demonstrated an impairment in quality of life by living with a WCD [18]. Forty-eight percent of patients with a WCD suffer from sleep disturbance and feel disabled in daily routine activities. Despite this, a WCD should be offered to motivated high-risk patients after individual consideration of the risk factors according to the guidelines [2, 9]. Additionally, improved and close monitoring of WCD patients by telemedicine might increase acceptance and quality of life in affected patients [19].

In our demonstrated case, the treatment of the electrical storm was performed according to current guideline recommendations and included a drug therapy: anti-arrhythmic pharmacotherapy was a combination of ß-blocker administration and amiodarone [20, 21]. As electrical storm is mostly observed in patients with ischemic heart disease and either is triggered by myocardial ischemia or occurs on the basis of scars as substrate; guidelines also recommend an urgent revascularization [20]. This, however, did not terminate the VT in our patient. Subsequent catheter ablation as part of our therapeutic approach in accordance with current recommendations finally stopped the recurrent episodes of electrical storm [9, 20, 21]. In theory, cardiac magnetic resonance imaging (MRI) to identify substrates for catheter ablation may increase ablation success. But despite the growing expertise on design changes for cardiac implants, the WCD is not suitable for MRI procedures [22].

In patients with an ICD, the termination of VT by antitachycardia pacing may also succeed, but this treatment option is not possible in WCD patients. The implantation of a cardiac resynchronization therapy defibrillator (CRT-D) and biventricular stimulation may also be an alternative therapeutic strategy [20]. In heart failure patients, unloading of the left ventricle by mechanical assist devices (for example Impella CP^®^) may stabilize the rhythm as well. A super-urgent orthotopic heart transplant should be considered as an ultima ratio in the absence of an effect with the other treatment options [20]. In general, ICD implantation is recommended for patients who survived sudden cardiac death [9].

## Conclusion

Here we report case of a patient who survived an electrical storm shortly after MI by the use of a WCD that delivered 17 appropriate shocks. Although there was no significant reduction of SCD in the VEST trial, WCD are a feasible option for arrhythmic protection and therefore recommended for high-risk patients until a final evaluation for an ICD is possible. It remains unclear how patients at increased risk of arrhythmic death can be identified in the early period after MI and before permanent ICD implantation is indicated.

## Data Availability

Data sharing is not applicable to this article because no data sets were generated or analyzed during the current study.

## Abbreviations

AED: Automated external defibrillator
CRT-D: Cardiac resynchronization therapy defibrillator
EP study: Electrophysiology study
ICD: Implantable cardioverter-defibrillator
IRIS: Immediate Risk Stratification Improves Survival
LAD: Left anterior descending artery
LAO view: Left anterior oblique view
MRI: Magnetic resonance imaging
SCD: Sudden cardiac death
RCA: Right coronary artery
RF: Radio-frequency
TIMI: Thrombolysis in Myocardial Ischemia Trial
VES: Ventricular extrasystole
VEST: Vest prevention of early sudden death trial
VT: Ventricular tachycardia
WCD: Wearable cardioverter defibrillator

## References

[1] Bardy GH, Lee KL, Mark DB, Poole JE, Packer DL, Sudden Cardiac Death in Heart Failure Trial (SCD-HeFT) Investigators (2005). Amiodarone or an ICD for congestive heart failure. N Engl J Med..

[2] Khan HM, Leslie SJ (2019). Risk factors for sudden cardiac death to determine high risk patients in specific patient populations that may benefit from a wearable defibrillator. World J Cardiol..

[3] Solomon SD, Zelenkofske S, McMurray JJ, Finn PV, Velazquez E, Ertl G, Harsanyi A, Rouleau JL, Maggioni A, Kober L, White H, Van de Werf F, Pieper K, Califf RM, Pfeffer MA, Valsartan in Acute Myocardial Infarction Trial (VALIANT) Investigators (2005). Sudden death in patients with myocardial infarction and left ventricular dysfunction, heart failure, or both. N Engl J Med..

[4] Moss AJ, Zareba W, Hall WJ, Klein H, Wilber DJ, Cannom DS, Multicenter Automatic Defibrillator Implantation Trial II Investigators (2002). Prophylactic implantation of a defibrillator in patients with myocardial infarction and reduced ejection fraction. N Engl J Med..

[5] Hohnloser SH, Kuck KH, Dorian P, Roberts RS, Hampton JR, Hatala R, Fain E, Gent M, Connolly SJ, DINAMIT Investigators (2004). Prophylactic use of an implantable cardioverter-defibrillator after acute myocardial infarction. N Engl J Med..

[6] Steinbeck G, Andresen D, Seidl K, Brachmann J, Hoffmann E, Wojciechowski D, Kornacewicz-Jach Z, Sredniawa B, Lupkovics G, Hofgärtner F, Lubinski A, Rosenqvist M, Habets A, Wegscheider K, Senges J, IRIS Investigators (2009). Defibrillator implantation early after myocardial infarction. N Engl J Med..

[7] Ponikowski P, Voors AA, Anker SD (2016). 2016 ESC Guidelines for the diagnosis and treatment of acute and chronic heart failure, The Task Force for the diagnosis and treatment of acute and chronic heart failure of the European Society of Cardiology (ESC) Developed with the special contribution of the Heart Failure Association (HFA) of the ESC. Eur Heart J.

[8] Anderson JL, Adams CD, Antman EM (2011). 2011 ACCF/AHA focused update in-corporated into the ACC/AHA 2007 guidelines for the management of patients with unstable angina/non-ST-elevation myocardial infarction: a report of the American College of Cardiology Foundation/American Heart Association Task Force on Practice Guidelines. Circulation.

[9] Priori SG, Blomström-Lundqvist C, Mazzanti A (2015). 2015 ESC Guidelines for the management of patients with ventricular arrhythmias and the prevention of sudden cardiac death, The Task Force for the Management of Patients with Ventricular Arrhythmias and the Prevention of Sudden Cardiac Death of the European Society of Cardiology (ESC). Europace.

[10] Feldmann AM, Klein H, Tchou P (2004). Use of a wearable defibrillator in terminating tachyarrhythmias in patients at high risk for sudden death: results of WEARIT/BIROAD. Pacing Clin Electrophysiol.

[11] Kutyifa V, Moss AJ, Klein H, Biton Y, McNitt S, MacKecknie B, Zareba W, Goldenberg I (2015). Use of the wearable cardioverter defibrillator in high-risk cardiac patients: data from the Prospective Registry of Patients Using the Wearable Cardioverter Defibrillator (WEARIT-II Registry). Circulation.

[12] Chung MK, Szymkiewicz SJ, Shao M (2010). Aggregate national experience with the wearable cardioverter-defibrillator: event rates, compliance, and survival. J Am Coll Cardiol..

[13] Olgin JE, Pletcher MJ, Vittinghoff E, Wranicz J, Malik R, Morin DP, Zweibel S, Buxton AE, Elayi CS, Chung EH, Rashba E, Borggrefe M, Hue TF, Maguire C, Lin F, Simon JA, Hulley S, Lee BK, VEST Investigators (2018). Wearable cardioverter-defibrillator after myocardial infarction. N Engl J Med..

[14] Bieber M, Werner RA, Tanai E, Hofmann U, Higuchi T, Schuh K, Heuschmann PU, Frantz S, Ritter O, Kraft P, Kleinschnitz C (2017). Stroke-induced chronic systolic dysfunction driven by sympathetic overactivity. Ann Neurol.

[15] Manufacturer information, LifeVest 4000, ZOLL, Pittsburgh, Pennsylvania, USA, https://lifevest.zoll.com/de/patients/living-with-lifevest. Accessed 24 March 2021.

[16] Duncker D, Veltmann C (2018). Role of the wearable defibrillator in newly diagnosed heart failure. Curr Heart Fail Rep..

[17] Olgin JE, Lee BK, Vittinghoff E, Morin DP, Zweibel S, Rashba E, Chung EH, Borggrefe M, Hulley S, Lin F, Hue TF, Pletscher MJ (2020). Impact of wearable cardioverter-defibrillator compliance on outcomes in the VEST trial: as-treated and per-protocol analyses. J Cardiovasc Electrophysiol..

[18] Lackermair K, Schuhmann CG, Kubieniec M, Riesinger LM, Klier I, Stocker TJ, Kääb S, Estner HL, Fichtner S (2018). Impairment of quality of life among patients with wearable cardioverter defibrillator therapy (LifeVest®): a preliminary study. Biomed Res Int..

[19] Ritter O, Bauer W (2006). Use of “IEGM Online” in ICD patients. Clin Res Cardiol..

[20] Gadula-Gacek E, Tajstra M, Gąsior M (2019). Electrical storm – still an extremely poor prognosis. Do these acute states of life-threatening arrhythmias require a multidirectional approach from the start?. Adv Interv Cardiol..

[21] Kontogiannis C, Tampakis K, Georgiopoulos G, Bartoletti S, Papageorgiou C, Anninos H, Kapelouzou A, Spartalis M, Paraskevaidis I, Chatzidou S (2019). Electrical storm: current evidence, clinical implications, and future perspectives. Curr Cardiol Reports.

[22] Nordbeck P, Fidler F, Friedrich MT, Weiss I, Warmuth M, Gensler D, Herold V, Geistert W, Jakob PM, Ertl G, Ritter O, Ladd ME, Bauer WR, Quick HH (2012). Reducing RF-related heating of cardiac pacemaker leads in MRI: implementation and experimental verification of practical design changes. Magn Reson Med..

